# Remote CI Fitting in Early Rehabilitation Phase: Preliminary Results

**DOI:** 10.1097/MAO.0000000000004648

**Published:** 2025-10-03

**Authors:** Nienke C. Homans, Hylke F.E. van der Toom, André Goedegebure, Jantien L. Vroegop

**Affiliations:** Department of Otorhinolaryngology and Head and Neck Surgery, Erasmus MC, Rotterdam, The Netherlands

**Keywords:** Cochlear implant, Cochlear rehabilitation, Otology, Rehabilitation, Remotefitting

## Abstract

**Objective:**

Rising cochlear implant (CI) candidate numbers and limited clinic resources challenge high-quality care. Remote programming via telemedicine offers a solution to enhance efficiency while maintaining patient satisfaction and outcomes. This study examined the feasibility of replacing an in-clinic fitting with a remote one during the rehabilitation phase.

**Patients:**

This study included 31 postlingually deafened adult CI users implanted at Erasmus Medical Center, Rotterdam, The Netherlands.

**Interventions:**

Recently, a new remote programming system allowed audiologists to program implants via the CI user’s smartphone app using Bluetooth connectivity. Twenty-one participants received standard in-clinic rehabilitation of 4 fitting appointments, while 10 patients underwent remote fitting for their third appointment.

**Main outcome measure(s):**

Auditory functioning and patient satisfaction was evaluated at 3 months postimplantation.

**Results:**

Participants in the remote group expressed high satisfaction with the process, with most of them recommending remote fitting to others. Most participants found the setup easy and appreciated the convenience of skipping an in-clinic visit. Technical performance was stable, with the exception of one CI user for whom it was not possible to establish the connection. No significant differences were observed between groups in free-field PTA thresholds, speech perception scores, or perceived auditory functioning (SSQ).

**Conclusions:**

Remote fitting proves to be a feasible alternative to in-clinic programming, yielding high patient satisfaction and similar auditory outcomes. It could optimize CI care by reducing clinic burden and improving accessibility, advancing future-proof CI health care. Further research with larger samples is needed to validate these findings and explore long-term effects. Incorporating streaming options and/or subtitles would enhance communication during the process.

## Objective

Cochlear implantation is a well-established treatment option for individuals with severe to profound hearing loss, marking a major shift in the management of this health concern. The high performance achieved by most cochlear implant (CI) users, combined with the rapid evolution of implant technology, has led to a significant expansion of the selection criteria for cochlear implantation.^1–3^ Consequently, the number of CI candidates is steadily increasing.^4^

In addition to the broader implantation criteria, the aging population is contributing to a growing number of individuals with hearing loss.^5^ Furthermore, despite the increasing use of CIs in recent years, their adoption within the group of potential candidates remains limited, with current prevalence estimates ranging from only 2% to 13%.^6–8^ Subsequently, a substantial group of individuals with severe hearing loss who could benefit from a cochlear implant remains untreated.

Given that CI rehabilitation programs typically involve multiple fitting appointments during the first year, exploring the potential of remote programming is essential to streamline care and support the scaling of CI implantation and rehabilitation.^9–11^

The primary objective of this study was to assess participants’ experiences with remote fitting through questionnaires. The secondary objective was to compare outcome measures between participants who received remote fitting and those who attended in-person sessions at 3 months postimplantation, focusing on free-field tone audiometry, speech perception scores, and auditory functioning questionnaire results.

## Patients

The study was conducted at the CI center of the *[blinded for review]*.

**Figure 1:**
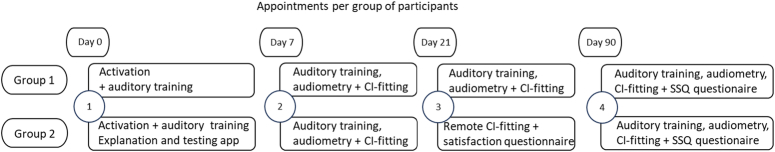
The rehabilitation process of both groups. Group 1 received the traditional fitting procedure, and group 2 received the remote fitting procedure.

The study population consisted of adult CI users aged 18 years and older who received a cochlear implant from Advanced Bionics. Two groups of participants were included in the study (Fig. 1).

1. Twenty-one CI users who were fitted using the traditional in-clinic method.
2. Ten CI users who received remote fitting during the third appointment of the rehabilitation phase.

No significant differences in baseline characteristics were observed between the 2 groups (Mann-Whitney *U* test, Table 2).

### Procedure standard in-clinic rehabilitation process

The subjects in group 1, who received the standard in-clinic rehabilitation process, followed the typical rehabilitation schedule, which included the initial activation and follow-up appointments at 1 week, 3 weeks, and 3 months post activation. During all visits, patients were seen by an audiologist and by a speech and language therapist. At the 3-month visit, an evaluation was conducted that included free field tone and speech audiometry to assess hearing outcomes.

### Procedure remote fitting process

Advanced Bionics (AB) (California, USA) introduced a new remote programming solution in 2023. This system integrates the standard Target CI fitting software for clinicians with the AB Remote Support smartphone app for cochlear implant users. The subjects in the remote programming group (group 2) received an explanation about the app during the initial activation or the follow-up visit in the clinic. Up to the third appointment, they followed the standard rehabilitation protocol. The third appointment (3 wk postactivation) was scheduled as a remote fitting session with the CI audiologist, without involvement of the speech and language therapist, to simplify logistics. This decision was also made because audiometry, typically conducted during the therapist’s session, cannot be performed remotely (Fig. 1). If CI users requested additional training exercises during this remote session, these were provided by the speech and language therapist afterwards. No audiometric evaluation was conducted during the remote session, in contrast to the in-clinic visits. The same programming procedure used during the in-clinic visits was applied during the remote fitting. In all remote fittings, a partner, child of the parent, was present to assist with communication with the audiologist if necessary. This also allowed a brief conversation together in a familiar environment to test the sound quality after the CI was adjusted. At the 3-month visit, the same evaluation was conducted as in the standard care process.

## Main outcome measures

### Audiometric evaluation

The audiometric evaluation consisted of free-field tone audiometry and speech audiometry. Free-field audiometry was performed with warble tones at standard frequencies, and the pure-tone average (PTA) for 500, 1000, 2000, and 4000 Hz was calculated.

To assess speech perception in quiet with the CI, the standard Dutch speech test used in clinical practice by the Dutch Society of Audiology^12^ was employed. This test consists of phonetically balanced monosyllabic (consonant–vowel–consonant) word lists, presented at 55 and 65 dB SPL.

### Questionnaires

For participants in both groups during the 3-month appointment, self-reported hearing ability was assessed with the Speech, Spatial, and Qualities of hearing scale (SSQ) by Gatehouse et al.^13^ We used the Dutch version 3.2.1 (2007), which is also available in a shortened form with 17 questions divided into 3 domains.^14^ The average score was calculated for each of the 3 domains. For the remote programming group, participants were asked to complete a subsequent set of questions to assess their experience with the remote fitting session. See Table 1 for these questions.

**Table 1:** Questionnaire and the answers

Questions	Answer options	(n)
1. How difficult/easy was it to connect with the audiologist?	Very difficult / not successful	1
	Difficult	0
	Neutral	0
	Easy	3
	Very easy	6
2. Was there contact with the audiologist throughout the entire session?	Yes, video and audio	9
	Yes, video only	0
	Yes, audio only	0
		
	No, sometimes no contact	1
3. How did you experience the online contact with the audiologist?	Very unpleasant	0
	Unpleasant	0
	Neutral	0
	Positive	4
	Very positive	5
4. Do you find this contact important?	Not important at all	0
	Not very important	0
	Neutral	0
	Important	6
	Very important/essential	3
5. How did you experience the adjustment of the CI?	Very unpleasant	0
	Unpleasant	0
	Neutral	1
	Positive	5
	Very positive	3
6. Was the session comparable to a fitting session in the clinic?	Yes, completely	2
	Some differences	2
	Different but equally effective	4
	Different, and I prefer the hospital	1
	Fittings should not be done at home	0
7. Would you like to have your CI adjusted this way again?	Definitely not	0
	Preferably not	1
	Neutral	1
	Probably yes	1
	Definitely yes/without hesitation	6
8. You had one less appointment with the speech therapist. How did you experience that?	Very unpleasant	1
	Unpleasant	0
	Neutral	4
	Positive	1
	Very positive / not missed	3
9. Instead of an in-clinic check-up, would you prefer an online CI check-up in the future?	Definitely not	1
	Preferably not	0
	Neutral	3
	Probably yes	3
	Definitely yes / without hesitation	3
10. Would you recommend the online adjustment of the CI to others?	Definitely not	1
	I don’t think so	0
	Neutral	0
	Probably yes	4
	Absolutely / without hesitation	4
11. In conclusion, how did you feel about the remote appointment?	Very unpleasant	1
	Unpleasant	1
	Neutral	0
	Positive	4
	Very positive	4

### Data analysis

The data were examined with SPSS (v28). Due to the non-normal distribution of most outcome data, nonparametric tests were employed for the data analysis. The Mann-Whitney test was used for independent comparisons between the 2 groups. A *P*-value <0.05 was considered statistically significant.

## Results

### Participant experiences

Table 1 shows the results of the questionnaire in which the participants’ experience with the remote fitting sessions was assessed. Of the 10 participants in the remote fitting group, 8 were positive to very positive about the experience. One participant could not complete the fitting due to connection issues.

Eight out of 9 would likely recommend remote fitting. Six participants noted it was harder to understand the audiologist remotely, though they still found the fitting equally effective.

Opinions on cancelling the speech therapist appointment were mixed: 4 were positive, 1 preferred to keep it, and 4 were neutral.

All participants were positive about the fitting and contact with the audiologist. Technical performance was stable in all but one session, and most found the setup easy. Some mentioned that a hearing partner helped overcome communication challenges.

### Auditory functioning

**Figure 2:**
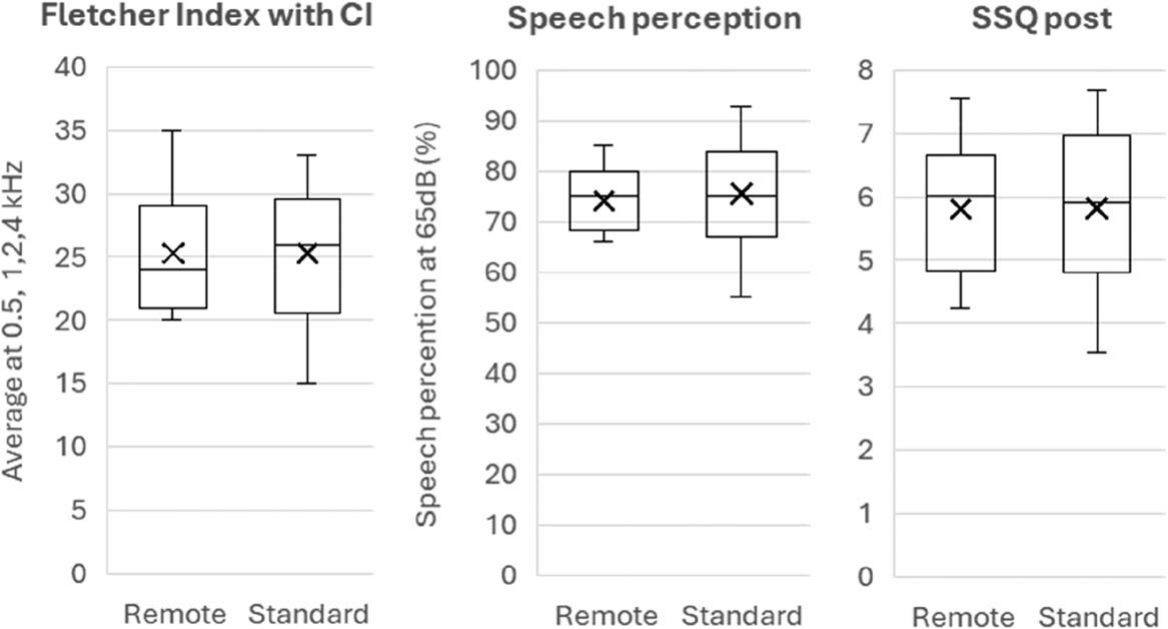
The box-whisker plot demonstrates the outcome measures for the remote and standard fitting groups. Boxes represent the median (horizontal line), lower and upper quartiles (ends of boxes), and minimum and maximum values (ends of whiskers). The left panel illustrates the average hearing thresholds with a cochlear implant (frequencies: 0.5, 1, 2, and 4 kHz). The middle panel presents the average phoneme recognition score for speech perception at 65 dB SPL, expressed as a percentage. The right panel depicts the outcomes of the average score of the Speech, Spatial, and Qualities of Hearing Scale (SSQ_17_).

At the 3-month evaluation of the CI rehabilitation phase, no significant differences in free field PTA and speech perception performance at 55 and 65 dB SPL were found between the 2 groups. Similarly, no differences in auditory functioning, as assessed by the SSQ or its sub-domains, were observed between the 2 groups (Mann-Whitney *U* test), as shown in Table 2 and Figure 2.

**Table 2:** Baseline characteristics and postimplantation scores at 3-month evaluation

	Standard fitting [mean(SD)]	Remote fitting [mean (SD)]	*P*
Age	68 (15)	65 (14)	0.492
PTA_preop_ (dB SPL)	96 (15)	88 (8)	0.124
PTA_CI_ (dB HL)	25 (5)	25 (5)	0.894
Speech perception at 65 dBSPL_preop_ (%)	32 (28)	45 (25)	0.233
Speech perception at 55 dBSPL_preop_ (%)	25 (26)	47 (19)	0.154
Speech perception at 65 dBSPLCI (dB SPL)	76 (11)	74 (7)	0.533
Speech perception at 55 dBSPLCI (dB SPL)	69 (15)	72 (17)	0.790
SSQ_total score_	5.8 (1.3)	5.8 (1.1)	0.901
SSQ_speech_	5.9 (1.5)	6.6 (1.0)	0.234
SSQ_spatial_	5.2 (1.6)	4.3 (2.3)	0.318
SSQ_quality_	6.1 (1.4)	5.6 (1.2)	0.534

dB SPL indicates decibel sound pressure level; PTA, pure tone average; SD, standard deviation.

### Session duration

The audiologist spent an average of 25 to 35 minutes on each remote fitting session, with a maximum duration of 45 minutes.

## Discussion

Remote programming solutions have the potential to make the CI rehabilitation process more efficient, which is highly warranted in the context of an increasing demand for CI care and limited resources. Our findings show that patient satisfaction was very high in the remote fitting group, with auditory outcomes comparable to those of the traditional rehabilitation group.

The vast majority of participants were very pleased and satisfied with the online fitting process. The communication was perceived as positive, and many participants specifically mentioned that not having to travel to the hospital was a significant advantage. However, some concerns were raised regarding the app's functionality that should be addressed.

- Lack of subtitles.
- Lack of direct sound streaming to the cochlear implant during the fitting process.
- A feature for measuring hearing thresholds should be available within the application.

By not scheduling a speech therapy appointment before the remote fitting, 45 minutes of speech therapist time was saved per patient during the rehabilitation process (first 4 appointments). In addition, the remote fitting sessions often took less time than the scheduled, similar to those in the clinic, 45 minutes, with an average duration of about 30 minutes. No time was spent on bimodal hearing or assistive listening devices during these remote fitting appointments, which may have shortened their duration. Together, this could result in a total time savings of ∼1 hour per patient for this remote appointment.

Building on these advancements, future studies could explore the effectiveness of both remote rehabilitation tools and remote fitting methods during different phases of the rehabilitation process and across a wider range of populations, such as pediatric patients. In addition, it would be valuable to investigate the extent of adjustments in the C-level and T-level during remote fitting. We demonstrated that remote fitting might be effectively applied even during the initial rehabilitation phase (within 1 to 2 mo), suggesting that further exploration of expanding remote fitting as an alternative to in-clinic visits is warranted.

## Conclusion

Remote fitting proved to be a feasible alternative to one of the in-clinic programming sessions during the early rehabilitation phase. It received high levels of patient satisfaction and maintained comparable auditory outcomes.
